# Liver resection for non-colorectal metastases

**DOI:** 10.1007/s10353-018-0528-y

**Published:** 2018-04-25

**Authors:** Christoph Schwarz, Klaus Kaczirek, Martin Bodingbauer

**Affiliations:** Department of Surgery, Medical University of Vienna, Währinger Gürtel 18–20, 1090 Vienna, Austria

**Keywords:** Liver metastases, Surgical technical improvement, Liver resection, Oncological prognosis, Oncological surgical treatment

## Abstract

Whereas liver resection for colorectal metastasis has become standard of care, hepatectomy in patients with non-colorectal metastases remains controversial, mainly due to a heterogeneous tumor biology and missing data from prospective trials. This review aims at giving an overview about the indications and limits of liver surgery in patients with an advanced disease of a non-colorectal malignancy. Even though prospective trials are largely missing, results from retrospective studies indicate a survival benefit for liver resection in selected patients. Thus, in metastasized patients, treatment strategies should be developed in a multidisciplinary tumor board including an experienced liver surgeon.

## Introduction

The liver is a frequent site for metastases of various kinds of malignancies. Whereas historically, metastasized malignancies have had a dismal outcome and limited treatment options, refinement of surgical techniques and advances in systemic and locoregional oncological therapy have pushed the indications for liver resection (LR) towards a more aggressive approach, where hepatectomy is still a potential option even in patients with advanced cancer [1].

Whereas liver resection for colorectal metastasis has been widely accepted, resulting in a substantially improved patient survival, hepatectomy for non-colorectal metastases remains controversial and is still under debate, which is mainly attributed to heterogeneous tumor subtypes.

This review aims at giving an overview about the indications and limits of LR in non-colorectal liver metastasis.

## Breast cancer (BRE)

After metastasizing in lymph nodes and lungs, the liver represents the third most common site for metastases of breast cancer [2]. Even though treatment strategies of metastasized breast cancer include systemic therapy, the use of liver resection has gained more and more acknowledgement. Only small retrospective studies investigating the impact of LR on survival for breast cancer metastasis have been published. However, a survival benefit has been suggested throughout the studies [3, 4].

Recently it has been shown in a cost-effectiveness study that liver resection for breast cancer metastases is cost-effective compared to systemic therapy alone, mainly in estrogen receptor-positive patients [5]. Currently, 5‑year overall survival is reported to range between 27 and 53%, depending on patient- and tumor-related factors [6]. Prognostic factors determining the outcome are response to preoperative chemotherapy, resection margin, and re-liver resection [4] or hormone receptor status [7, 8]. Especially patients with estrogen receptor-positive status might benefit from liver resection.

Mariani et al. proposed the indication for LR to be resectable liver metastasis (≤4 metastases), stable disease or disease responding on medical treatment, good patient performance status, and the only allowable extrahepatic disease being stable bone metastasis [9].

## Gastrointestinal (GI)

Due to the venous drainage via the portal vein, most gastrointestinal malignancies metastasize primarily to the liver. GI malignancies mainly include esophageal cancer, gastric adenocarcinoma, and gastrointestinal stromal tumors (GIST).

Liver metastases in patients with gastric cancer are diagnosed in up to 2–14% synchronously [10] and in 37% metachronously after gastric resection [11]. A recent meta-analysis suggests improved survival rates following liver resection for gastric adenocarcinoma (hazard ratio, HR: 0.50; [12]). Negative risk factors include multiple liver metastasis [13, 14], the size of metastases, and serosal invasion of the primary gastric cancer [14]. Thus, currently, the resection of small numbers metastases is recommended in combination with perioperative chemotherapy [15].

GISTs are the most common mesenchymal tumors of the gastrointestinal tract and it is estimated that 47% are synchronously metastasized [16], with the liver being the most frequent site of metastasis [17]. After LR, 5‑year survival rates range between 70 and 76% [18, 19], with complete resection (R0) being a significant prognostic factor for long-term survival [19]. Notably, preoperative tyrosine kinase inhibitor (TKI) therapy is suggested to be connected with a benefit in survival [19–21]. Thus, LR should only be recommended in patients under perioperative TKI therapy who are responding on medical treatment.

Only small case series exist regarding LR for metastasis of esophageal cancer. Even though most studies suggest LR is feasible in selected patients, 5‑year survival of patients after LR is less than 15%, and therefore currently not generally recommended [18].

## Melanoma (MEL)

There are two subgroups of melanoma: cutaneous and ocular melanoma, both with distinct pathological features. Because of the absence of lymphatic tissue in the uveal tract, ocular melanomas mainly metastasize hematogenously, with the liver being the main affected organ. In contrast, cutaneous melanoma rarely metastasizes to the liver but rather to the lungs, lymph nodes, or soft tissues [22]. From retrospective studies, 5‑year survival after LR is between 7 and 20%, with a median survival of 14–28 months [6]. Notably, R0 resection should be the aim [23, 24].

Overall, studies suggest a survival benefit for surgery in resectable patients; however, LR should be performed with concomitant systemic therapy, as recurrence is common after hepatectomy.

## Pancreato-biliary (PB)

Pancreatic ductal adenocarcinoma (PDAC) is one of the most fatal malignancies, with an increasing incidence over the past decades [25, 26]. It has been suggested that the metastases of pancreatic cancer occur relatively late after the initial development of the primary tumor [27]. Due to limited systemic treatment options, it is generally not recommended to resect metastases of PDAC [28, 29]. However, some case series have suggested that liver resection might be a potential option in selected patients with synchronous or even metachronous disease [30, 31]. Five-year survival after LR for PDAC metastasis is low and ranges between 5.8 and 20% [18, 30, 32]. Thus, currently, LR for patients with metastasized PDAC should not be performed in all patients but rather only in patients with a good tumor biology (response to chemotherapy, long interval between primary resection and appearance of liver metastases in patients with metachronous disease).

## Genitourinary (GU)

Genitourinary tumors mainly include renal cell carcinomas (RCC), gynecological carcinomas especially ovarian cancer, and testicular malignancies.

For patients with RCC undergoing LR, overall 5‑year survival rates of 38–62% were reported, with a median survival of 33–142 months [33–35]. Risk factors for poor outcome are extrahepatic disease, a short disease-free interval [35], lymph node metastases, poor performance status, high-grade tumors [34], and a positive resection margin [33].

5-year overall survival rates of 30–51% in patients with ovarian cancer were described in several studies with median survival times of 26–98 months [36, 37]. Risk factors for worse prognosis are a short interval from diagnosis of primary disease to metastasis [37], positive resection margins, pre-operative ascites, and bilobar disease [36].

Only limited data exist for hepatectomy for testicular carcinoma, but the combination of chemotherapy and liver surgery demonstrated improved outcome in patients who faced this treatment plan [38].

## Head and neck (HN)

Head and neck cancers include oral, nasopharyngeal, and laryngeal malignancies. These cancers are most frequently squamous cell carcinomas. In general, these patients experience poor prognosis following LR, with 5‑year survival rates of less than 15% [18]. Better outcome was reported for liver metastases from undifferentiated nasopharyngeal carcinomas with an overall survival rate of 40.2% [39].

## Thyroid cancer (TC)

Papillary and follicular thyroid carcinomas are known as differentiated thyroid carcinomas (DTC) and are among the most curable of cancers. Liver metastases from DTC are exceptionally rare; only a few cases are reported in the literature, but surgical resection should always be considered as this offers the best chance for prolonged survival [40].

## Neuroendocrine tumors (NET)

Neuroendocrine tumors often metastasize to the liver (50–75%). Surgical resection and transplantation have been offered in a curative intent, but palliative cytoreductive surgery can also be considered for disease control. In only few patients (7–15%) can a curative resection be realized [41]. The role of surgical intervention was studied to be the best approach for resectable liver metastases from NET in terms of prolonged survival and relief of symptoms, despite the high incidence of recurrence after surgery [42].

Hepatectomy has become a safe surgical procedure and resectability can more often be pushed to a further level also in advanced oncological states. In the context of a metastatic setting of an NET tumor, the surgical intention should be the removal of all NET metastases within the liver parenchyma with curative intent. Even in combination with non-resectable liver-directed treatments, including ablative techniques, transarterial chemoembolization (TACE), or internal radiation therapy (radioembolization). All these new treatments have been shown to be successful in slowing tumor progression and providing symptom relief [43, 44].

In general, well-differentiated, low- or intermediate-grade NETs have a relatively indolent behavior with slow progression and can be treated. Poorly differentiated tumors may exhibit highly aggressive behavior with rapid metastatic spread, and surgery therefore plays an insufficient treatment role in this setting.

Only a low level of recommendations exists to assist in the treatment of patients with neuroendocrine liver metastases. The efficacy of the NET management, either surgery alone or in combination with non-resectable treatments, remains unproven [45].

## Cancer of unknown primary (CUP)

A cancer of unknown primary is often associated with an unfortunate outcome, because patients are usually treated with non-standardized therapy. LR of metastases of a CUP can improve prognosis and is reported with a 5-year survival of 38% [18].

## Discussion

Liver surgery has become a valuable treatment option even for patients with advanced cancer. In combination with suitable neoadjuvant and adjuvant chemotherapy, local tumor control is the only chance to ensure long-term survival for patients with metastasized disease. While LR for colorectal or neuroendocrine metastasis has become standard of care today, hepatectomy for non-colorectal metastasis remains controversial, which is mainly attributed to a lack of prospective data due to a relatively low incidence, but also due to missing powerful chemotherapy options for most tumor types. However, the safety of hepatectomy has increased over the past decades [1] and oncological therapy has improved substantially. Therefore, aggressive treatment even of an advanced disease is gaining more and more acknowledgment.

Prospective trials are necessary to better characterize the role of liver resection embedded in a multidisciplinary treatment approach. Moreover, an adequate patient selection based on tumor biology and patient-related factors is important to further improve outcome after LR.

Liver resection is a suitable treatment for non-colorectal cancer liver metastases. It can offer acceptable long-term survival, comparable to colorectal cancer metastases. Patients with controlled systemic disease benefit from hepatic resection and the treatment of liver metastases should be considered regardless of the primary tumor site. For individual patients with controlled systemic disease, hepatic resection can offer appropriate survival rates and should be considered regardless of the primary tumor site. It should be part of the oncological treatment.
